# The Small GTPase Rab7 Regulates Antigen Processing in B Cells in a Possible Interplay with Autophagy Machinery

**DOI:** 10.3390/cells12212566

**Published:** 2023-11-02

**Authors:** Marika Runsala, Elina Kuokkanen, Eveliina Uski, Vid Šuštar, Meryem Özge Balci, Johanna Rajala, Vilma Paavola, Pieta K. Mattila

**Affiliations:** 1 Institute of Biomedicine, and MediCity Research Laboratories, University of Turku, 20014 Turku, Finland; 2 InFLAMES Research Flagship, University of Turku, 20014 Turku, Finland; 3 Turku Bioscience, University of Turku and Åbo Akademi University, 20520 Turku, Finland

**Keywords:** adaptive immune system, B cell activation, antigen processing, vesicle traffic, B cell receptor, BCR, MHCII, endosomes, lysosomes, autophagy, Rab7

## Abstract

In B cells, antigen processing and peptide-antigen (pAg) presentation is essential to ignite high-affinity antibody responses with the help of cognate T cells. B cells efficiently internalize and direct specific antigens for processing and loading onto MHCII. This critical step, which enables pAg presentation, occurs in MHCII compartments (MIICs) which possess the enzymatic machinery for pAg loading on MHCII. The intracellular transport systems that guide antigen and maintain this unique compartment remain enigmatic. Here, we probed the possible functional role of two known endosomal proteins, the Rab family small GTPases Rab7 and Rab9, that are both reported to colocalize with internalized antigen. As compared to Rab9, we found Rab7 to exhibit a higher overlap with antigen and MIIC components. Rab7 also showed a higher association with antigen degradation. The inhibition of Rab7 drastically decreased pAg presentation. Additionally, we detected the strong colocalization of perinuclearly clustered and presumably MIIC-associated antigen with autophagy protein LC3. When we pharmacologically inhibited autophagy, pAg presentation was inhibited. Together, our data promote Rab7 as an important regulator of antigen processing and, considering the previously reported functions of Rab7 in autophagy, this also raises the possibility of the involvement of autophagy-related machinery in this process.

## 1. Introduction

Our bodies are in a constant battle against infections. To survive in this combat, we are protected by the sophisticated system of immunity in which B lymphocytes play an essential role by mounting the humoral immune response. Before B cells can mature into memory B cells or antibody-producing plasma cells, they have to undergo several cell biological changes. The specific antigen engagement of the B cell receptors (BCRs) triggers a strong, multibranched signaling cascade that leads to various cellular reactions ultimately inducing transcriptional re-profiling [1,2]. An important branch in B cell activation is the process of internalizing the antigen–BCR complexes and processing them into the intracellular compartments. The antigen is directed to the so-called major histocompatibility complex class II (MHCII) compartment (MIIC), where the antigen is processed into peptides (pAg) and loaded on MHCII for presentation. MIICs are acidic multivesicular compartments which contain MHCII and the critical accessory proteins to control pAg loading onto MHCII. The freshly formed pAg-MHCII complex is then transported to the plasma membrane to obtain critical help from cognate T helper (T_H_) cells. The interaction with T_H_ cells is critical to drive the formation of the germinal centers and B cell differentiation into high-affinity antibody-producing plasma cells and memory cells [1,3,4,5,6,7]. At the same time, pAg-MHCII presentation also stimulates the cognate T_H_ cells to orchestrate other branches of the immune system, and to generate CD4+ T cell memory [2,8]. Other antigen-presenting cells (APCs) of the immune system, particularly dendritic cells and macrophages, present peptides from unspecific extracellular complexes or phagocytosed material, while B cells are unique in their supreme efficiency in presenting peptides specific to their B cell antigen receptor, BCR [4,9]. Most of the studies on pAg loading and MIIC have been performed in these professional APCs, that do not undergo such antigen-specific presentation as B cells. Although the antigen processing and presentation by B cells is an important part of the adaptive immune response, the details of this process remain poorly understood.

In the antigen-processing compartments, also MHCII undergoes maturation. To protect MHCII from low affinity antigen binding, it is first enveloped with an invariant chain (Ii) that has to be degraded to allow pAg loading. This is a carefully guided process where Ii is clipped with CathepsinS (CatS) into a short tail called CLIP that protects the MHCII pAg binding groove until a proper pAg is inserted into the groove [1,10,11,12]. The exchange of CLIP with pAg is promoted by yet another molecule, DM (H2-M in mice), which twists MHCII such that the pAg binding groove stays open for pAg insertion [13,14]. While the biochemical details of antigen loading are quite well understood, and are likely occurring similarly in B cells and other APCs, much less is known about how the cargo is directed to MIICs and how the vesicular antigen processing machinery is created and maintained. To understand the regulation of the antigen processing in B cells, detailed studies on the antigen vesicle trafficking are required.

The general understanding of the heterogeneous nature of the intracellular vesicle trafficking pathways has been gained with modern microscopy techniques, and by the identification of a multitude of different vesicular carriers, like the Rab family of small GTPases [15,16,17,18,19] and vesicle sorting complexes such as retromer and retriever [20,21,22]. The research has revealed high dynamics within the endolysosome-related organelles and the enormous diversity in the compartments, varying even more between different cell types and cellular conditions [23]. In our recent studies, we identified remarkable endolysosomal heterogeneity in B cells. Internalized Ag is gathered to a perinuclear cluster accompanied with both early and late endosomal markers, suggesting for atypical compartments with mixed endosomal identity [24]. We further demonstrated that antigen, together with cell surface-derived MCHII, was directed to similarly heterogeneous but peripherally located specialized endosomes immediately after activation. These peripheral endosomes, named early MIIC (eMIIC), bear potential for kick-starting the antigen processing and pAg presentation already before gathering the classical MIICs in the perinuclear area, which occurs in approximately 30–60 min after the antigen encounter [24].

To better understand the mechanisms of antigen transport, here, we seek to further investigate the regulation of the antigen processing pathway by the Rab GTPases, which are known for their ability to coordinate the vesicle compartment identities and transport events [15,16,17,18,19]. Particularly, we are interested in the antigen gathering to the perinuclear area, where the long-term processing for peptide-presentation would occur. Among the canonical Rab proteins, Rab7 and Rab9 are typically associated with late endosomes and we have also reported that both of them associate with Ag vesicles in B cells [24]. Not much has been reported about the functional role of Rab-proteins in Ag-processing but, interestingly, Rab7 has been shown to respond to BCR activation [25] and it is involved in activation-induced cytidine deaminase expression and class-switch recombination (CSR) [26,27,28]. Not much is known about the role of Rab9 in B cells.

In this report, we show that, while the two predominantly late endosomal Rab proteins, Rab7 and Rab9, were markedly colocalized in B cells, they showed differential correlation with antigen processing. By utilizing microscopy, cells expressing mutated Rab7 and Rab9, as well as cognate B–T cells lines with immunoassays, we suggest that Rab7 plays an important role in antigen processing and pAg presentation. Additionally, upon its perinuclear accumulation, we found antigen to strongly correlate with autophagy-linked regulator LC3 and propose a connection between antigen processing and autophagy.

## 2. Materials and Methods

### 2.1. Cells and Transfections

A20 mouse lymphoma cells stably expressing a hen egg lysozyme (HEL)-specific IgM BCR (D1.3) [29] and 1E5T cells, specific for the HEL^108–116^ peptide presented in the MHCII *I-A^d^* [30], were kind gifts from Prof. Facundo Batista (the Ragon Institute of MGH, MIT and Harvard, MA, USA). Cells were cultured in complete RPMI (cRPMI; RPMI 1640 with 2.05 mM L-glutamine supplemented with 10% fetal calf serum (FCS), 50 μM β-mercaptoethanol, 4 mM L-glutamine, 10 mM HEPES, and 100 U/mL penicillin/streptomycin).

MD4 mice (C57BL/6-Tg(IghelMD4)4Ccg/J) (the Jackson Laboratory) for primary B cell isolation were maintained under specific-pathogen-free conditions and culled at the age of 8–12 weeks. Animal maintenance was approved by the Ethical Committee for Animal Experimentation in Finland and adhered to the Finnish Act on Animal Experimentation (62/2006; animal license numbers: 7574/04.10.07/2014 KEK/2014-1407-Mattila, 10727/2018).

A20 D1.3 cells were transfected as previously described [31]. Briefly, 4 × 10^6^ cells were transfected with 4 µg (or 2 µg + 2 µg) of plasmid(s) in 0.2 cm gap electroporation cuvettes, in 180 µL of 2S transfection buffer (5 mM KCl, 15 mM MgCl_2_, 15 mM HEPES, 50 mM sodium succinate, 180 mMNa2HPO4/NaH_2_PO_4_ pH 7.2) with AMAXA electroporator (program X-005, Biosystem). Cells were immediately transferred into 2 mL of cRPMI supplemented with 10% FCS and left in 5% CO_2_ at 37 °C to recover for 3–4 h before performing experiments.
**Plasmids**
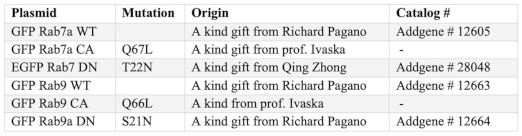
**Antibodies**
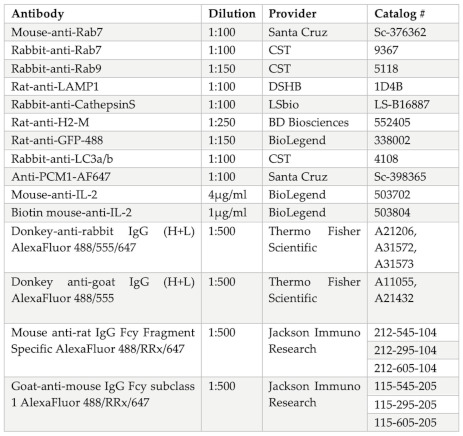


### 2.2. Sample Preparation for Confocal Microscopy

A20D1.3 B cells were activated, or not, with 10 µg/mL of anti-mouse-IgM-F(ab)’_2_ fragments labelled with Alexa Fluor 647 or -Rhodamine Red-X (Jackson ImmunoResearch 715-606-020/715-296-020) in 10% FCS-PBS. Cells were activated for the desired time points in 5% CO_2_, 37 °C, on the microscopy slides.

Either 12-well PTFE diagnostic slides (Thermo Fisher Scientific, Waltham, MA, USA, 10028210) or 8-well IBIDI uncoated µ-slides (IBIDI, 80806) were used. To allow cells to adhere on the 12-well slides, the slides were coated with 1 µg/mL fibronectin at RT for 30 min. The cells were then seeded on the slides simultaneously with soluble surrogate antigen activation. Cells were fixed with 4% PFA 10 min RT and permeabilized in blocking buffer (5% donkey serum, 0.3% Triton X-100 in PBS) for 1 h at RT. After blocking, samples were stained with primary antibodies at 4 °C overnight in staining buffer (1% BSA, 0.3% Triton X-100).

Eight-well slides were coated with 22.4 µg/mL CellTak in pH 8.0 for 1 h at RT and then air-dried. Cells were seeded on the slides for activation, permeabilized with 50:50 acetone–methanol at −20 °C for 20 min, and permeabilized with acetone at −20 °C for 5 min. Finally, the cells were blocked with 5% donkey serum in PBS for 1 h at RT and stained with primary antibodies in staining buffer (1% BSA in PBS) at 4 °C overnight.

Primary antibody staining was followed by washing (PBS) and incubation with secondary antibodies in PBS at RT for 1 h. Samples were mounted using FluoroMount g containing DAPI (Thermo Fisher Scientific, 00495952).

For live cell imaging, MatTek dishes were coated with 1 µg/mL fibronectin at RT for 30 min, washed, and 400,000 cells (in PBS supplemented with 10% FCS) were placed on the dish to settle. Cells were placed in the pre-heated (37 °C) SDCM chamber and imaged with continuous capturing on Photometrics Evolve 10 MHz Back Illuminated EMCCD (512 × 512 pixels, 1 × 1 binning).

### 2.3. Image Acquisition and Processing

Spinning disk confocal microscopy (SDCM) images were acquired using a 3i CSU-W1 spinning disk equipped with 405, 488, 561, and 640 nm laser lines and 510–540, 580–654, and 672–712 nm filters and 63× Zeiss Plan-Apochromat objective. Hamamatsu sCMOS Orca Flash4 v2|C11440-22CU (2048 × 2048 pixels, 1 × 1 binning) was used to image the fixed samples. The Photometrics Evolve 10 MHz Back Illuminated EMCCD (512 × 512 pixels, 1 × 1 binning) camera was used to image DQ-Ova data.

All SDCM images were deconvoluted with Huygens Essential v16.10 (Scientific Volume Imaging, Hilversum, The Netherlands, http://svi.nl, accessed 13 June 2019), using the CMLE algorithm, with a signal-to-noise ratio of 20 and 40 iterations.

### 2.4. Transmission Electron Microscopy (TEM) Sample Preparation and Acquisition

TEM was used to study the antigen internalization and intracellular structures of activated A20D1.3 cells as in [24]. Briefly, A20D1.3 cells were activated with α-IgM-Fab_2_-647 and 6 nm colloidal-gold conjugated goat anti-mouse IgM (1:650, Jackson ImmunoResearch, 115-195-075), with a combined concentration of 20 µg/mL, in imaging buffer (10% FBS in PBS, 5.5 mM D-Glucose, 0.5 mM CaCl_2_, and 0.2 mM MgCl_2_). Activated cells were placed on 4 µg/mL fibronectin-coated glass coverslips (thickness #1) for 15 or 75 min, fixed with 2% glutaraldehyde (EM grade, G7651, Sigma-Aldirch, Burlington, VT, USA) in 0.1 M sodium cacodylate buffer with pH 7.4, for 30 min at RT, and washed twice with 0.1 M sodium cacodylate buffer, with pH 7.4.

The samples were processed for EM as described in (Jokitalo et al., 2001). In short, 60 nm thick sections parallel to the cover slip were cut using a Leica EM Ultracut UC7 ultramicrome (Leica Mikrosysteme GmbH, Vienna, Austria). The electron micrographs were post stained with uranyl acetate and lead citrate, and imaged with a Jeol JEM 1400 transmission electron microscope (Jeol Ltd., Tokyo, Japan) equipped with a bottom mounted CCD camera (Orius SC 1000B, Gatan Inc., Pleasanton, CA, USA) and Jeol JEM-1400 Plus equipped with an OSIS Quemesa bottom-mounted CCD camera (EMSIS, Muenster, Germany), both operating at 80 kV.

### 2.5. Structured Illumination Microscopy (SIM) Acquisition

For SIM, the cells were seeded in 0.5% FCS-PBS on 4 µg/mL fibronectin-coated MatTek dishes (35 mm glass-bottom dishes, TIRFM quality, P35G-0.170-14-C, MatTek Corporation, Ashland, MA, USA) and activated for desired times in 5% CO_2_, +37 °C and processed for immunofluorescence as above. The samples were mounted with Vectashield (Vector Laboratories, Newark, CA, USA) before imaging with the DeltaVision OMX super-resolution system.

DeltaVision OMX V4 was equipped with 60× SIM Olympus Plan Apo N objective (NA 1.42) and 405, 445, 488, 514, 568, and 642 nm lasers and corresponding filters for DAPI, CFP, Alexa488, YFP, Alexa 568, and Cy5 with the optical sectioning of 0.125 µm steps. Images were captured with three PCO Edge Front illuminated sCMOS (1024 × 1024 pixels) and processed with OMX Acquisition v3.70 and softWoRx Deconvolution v7.0.0.

### 2.6. DQ-Ova Assay

DQ-Ova proteolysis reporter DQ-Ovalbumin (Thermo Fisher Scientific, D12053) was biotinylated in-house with EZ-Link Maleimide–PEG2–biotin (Thermo Fisher Scientific, 21901BID). HEL from (Sigma-Aldrich, #L6876) was biotinylated using EZ-Link™ Sulfo-NHS-LC-LC-Biotin (Thermo Fisher Scientific, 21338). A20 D1.3 cells were incubated with 10 μg/mL Biotin-HEL for 10 min on ice, washed with PBS, and incubated with 1:1000 dilution of either Alexa Fluor 633 conjugated Streptavidin or pure Streptavidin on ice for 5 min. Biotin–streptavidin-treated B cells were again washed with PBS and stained with 10 μg/mL biotinylated DQ-Ova on ice for 5 min, and washed. Consequently, BCRs were linked with biotin(HEL)–streptavidin(633/pure)–Biotin(DQ-Ova). Cells were transferred in 12-well slides coated with fibronectin and allowed to activate by shifting to 5% CO_2_ at 37 °C for 1 h to allow the internalization of the probe-linked antigen. Samples were fixed with 4% PFA, stained as above, and processed for microscopy.

### 2.7. Antigen Presentation Measured by ELISA

A20 D1.3 cells were incubated with 5 µg/mL of HEL for 1 h at 37 °C in cRPMI. After 1 h, cells were washed and resuspended in cRPMI. Activated B cells were mixed with 1E5 T cells (ratio 2:1) and placed in 5% CO_2_ at 37 °C for 17 h. Then, 1 µM Wortmannin (SigmaAldrich, W1628) was added for the time of B cell activation only and the samples were washed before adding the T cells. Moreover, 60 µM CID1067700 (Merck, #SML0545) [26] was added for the full course of the experiment due to the fast reversible nature of the drug. Secretion of IL-2 was measured with ELISA. The 96-well ELISA plates (Microlon 600, Bio-One 675061) were coated with anti-IL-2 for 1 h at 37 °C in 25 µL of PBS. Non-specific binding sites were blocked with blocking buffer (1% BSA-PBS) at 4 °C overnight. Additionally, 50 µL of the co-culture supernatant was added to each well for 1 h at 37 °C and 50 µL of biotin-conjugated anti-IL-2 was added on the plate for 1 h at RT. Finally, blocking buffer containing ExtrAvidin Alkaline Phosphatase (1:5000, Sigma-Aldrich, 043M4771) was added and the plate was incubated 1 h at RT. Plates were washed with 0.05% Tween-20–PBS between the steps. Finally, the wells were supplemented with 50 µL of pNPP (SIGMAFAST p-Nitrophenyl phosphatase tablets, Sigma-Aldrich^®^, N2770) and the optical density was measured at 405 nm (Multiscan, Thermo Scientific, SkanIt Software, vs. 3.1.0.4). Samples were run in triplicates. All the samples were normalized to the control samples.

### 2.8. Antigen Internalisation by Flow Cytometry

A20D1.3 cells were labeled on ice with biotinylated α-IgM (Southern Biotech) or house-made HEL-biotin in PBS, for 10 min. Stained cells were incubated at 37 °C and 5% CO_2_ desired time points. Internalization was followed by staining with streptavidin-633 on ice for 20 min. Cells were washed with PB and analyzed with BD LSR Fortessa, equipped with 405, 488, 561, and 640 lasers with corresponding filters, and a software BD FACSDiva™ v8. The acquired data were gated on single cells, expressing GFP when applicable. In the experiments with inhibitors, cells were incubated with the inhibitors together with F(ab’)_2_ first on ice for 30 min, and then the inhibitors were kept in the media during the normal continuation of the assay. The data were analyzed using FlowJo v10 (Tree Star).

### 2.9. Microscopy Data Analysis, Statistical Analysis, and Illustrations

Rab7 and Rab9 particle analysis was carried out on deconvoluted SDCM images with Huygens Essential version16.10 (Scientific Volume Imaging). The Rab7/Rab9 channel was used as the threshold for the particles such that the dimmest 5% of the channel intensities were considered as the background. The signals outside the threshold for the particles were cleared from the both channels, Rab7/Rab9 and DQ-Ova. After clearing, the DQ-Ova intensity was studied in the Rab7/Rab9 particles (mean DQ-Ova intensity). Then, DQ-Ova voxels were summed together and divided by the summed Rab7 or Rab9 voxels to provide the ratio of the DQ-Ova colocalizing the Rab voxels to all Rab voxels.

Colocalization analyses were performed with Huygens Essential version 16.10 (Scientific Volume Imaging), using optimized automatic thresholding. The analyses used included Manders overlap coefficient, Pearson’s correlation coefficient, and the overlap coefficient. The overlap coefficient is also known as the Szymkiewicz–Simpson coefficient, is defined as the size of the union of set A and set B over the size of the smaller set between A and B, and was chosen in the analyses of the vesicle marker colocalization due to the ignorance of this method of the intensity differences between the channels and samples.

Antigen vesicle analysis for volume, distance, and intensity was performed from deconvoluted images using batch processing in MATLAB R2018b (MathWorks) described in [24]. The DQ-Ova vesicle analysis was performed with particle analysis tools in Huygens Essential v16.10.

Statistical significances were calculated using an unpaired Student’s *t*-test assuming the normal distribution of the data. Statistical values are denoted as: * *p* < 0.05, ** *p* < 0.01, *** *p* < 0.001, **** *p* < 0.0001. Graphs were created in GraphPad Prism 6 (GraphPad Software, La Jolla, CA, USA). Figures were prepared using Inkscape v.092.2.

## 3. Results

### 3.1. Rab7 and Rab9 Largely Colocalize but Show Distinct Responses to Antigen Activation in B Cells

**Figure 1:**
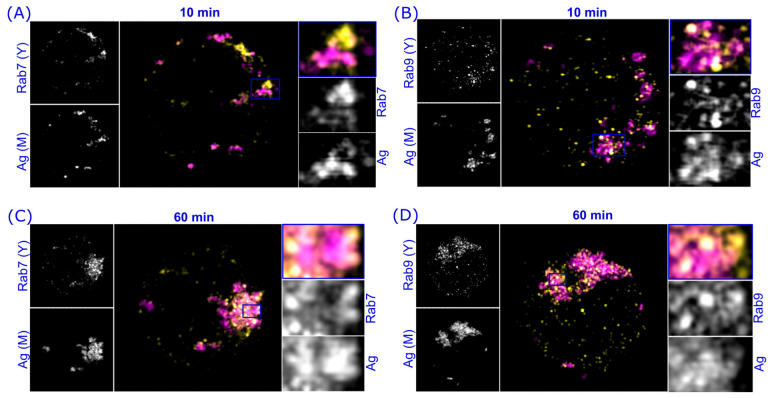
Rab7 and Rab9 in primary mouse B cells visualized with structured illumination microscopy (SIM). (**A**–**D**) Primary MD4 mouse B cells were activated for 10 (**A**,**B**) or 60 min (**C**,**D**) with α-IgM-647 (magenta) and processed for immunofluorescence analysis with anti-Rab7 ((**A**,**C**), yellow) or anti-Rab9 ((**B**,**D**), yellow) antibodies. The cells were imaged with 3D SIM. Stacked images of 13 planes with a step size of 0.125 µm, corresponding to 1.625 µm stack height, are shown. Images on the right are focused upon in the area marked with a blue box from the whole cell image in the middle. Image size in the images with whole cells (left and middle) 7 × 7 µm. Images are representative of 5–17 cells per condition.

In our previous work, we revealed the heterogenous colocalization of antigen with several endosomal vesicular markers at varying levels at different time points and subcellular locations [24]. With the aim of better understanding the regulation of antigen migration and targeting to the perinuclear MIIC, we focused on the two late endosomal trafficking proteins Rab7 and Rab9, that both show colocalization with antigen vesicles. We first wanted to verify the accumulation of Rab7 and Rab9 with an internalized antigen in primary B cells, as the antigen vesicle transport has previously been mostly studied in B cell lines. Due to the small size of the primary B cells, around 5 µm in diameter, the imaging of the small subcellar structures like endosomes is particularly challenging in them. To overcome this, we utilized the super-resolution imaging method structured illumination microscopy (SIM) and isolated the splenic B cells from the MD4 mouse strain where B cells express relatively high and homogenous levels of IgM BCR. We inspected the localization of endogenous Rab7 and Rab9 to visualize in higher detail how these late endosomal proteins accumulate with internalized antigen and the perinuclear antigen cluster over time. We activated the MD4 primary B cells for 10 or 60 min with fluorescent AlexaFluor647-labelled α-IgM antibody F(ab’)_2_ fragments (α-IgM-647) as surrogate antigen, proceeded for immunostainings with Rab7 or Rab9, and imaged the cells with SIM. With this approach, we were able to visualize the Rab7 and Rab9 vesicle pools in relation to antigen and detected the quite different organization of these two markers. While Rab7 appeared more concentrated on antigen both at early (10 min) and late (60 min) time points, Rab9 showed a more scattered pattern throughout the cells and more punctate colocalization with antigen (Figure 1). Upon antigen clustering to the perinuclear region in 60 min, both Rab7 and Rab9 showed the overall enrichment at the antigen-rich areas. However, Rab7 accumulated more clearly to the dense antigen cluster (Figure 1C), whereas Rab9 remained somewhat more dispersed (Figure 1D). This was also seen in the quantification where the overlap between Rab9 and Ag dropped in the 60 min time point compared to 10 min. The overlap between Rab7 and Ag stayed unchanged between 10 and 60 min (Figure S1A). This finding prompted a question of whether Rab7 and Rab9 decorated the same vesicles and co-operated in antigen processing, or whether they define different sets of vesicles and trafficking.

The most unambiguous way of determining whether the two vesicular markers decorate the same vesicle is to track their mobility in living cells. For the next experiments, we turned to cultured B cells to enable the transfection of fluorescently tagged Rab7 and Rab9. We transiently expressed both GFP-Rab7 and mCherry-Rab9 in a mouse B cell line A20 expressing the D1.3 IgM BCR (A20D1.3) and visualized the cells live to determine whether the two late endosomal proteins decorated the same vesicles. We followed non-activated and α-IgM-activated B cells with spinning disc confocal microscope (SDCM). We noticed that exogenously expressed Rab7 and Rab9 were typically found in the same endosomes in both conditions (Figure S1A,B, Videos S1 and S2). However, as generally accepted and also seen in our videos, the overexpression of Rab7 and Rab9 enlarges Rab7/9^+^ vesicles, suggesting alterations in the endosomal characteristics. Thus, Rab7–Rab9 colocalization might be different in non-transfected cells.

**Figure 2:**
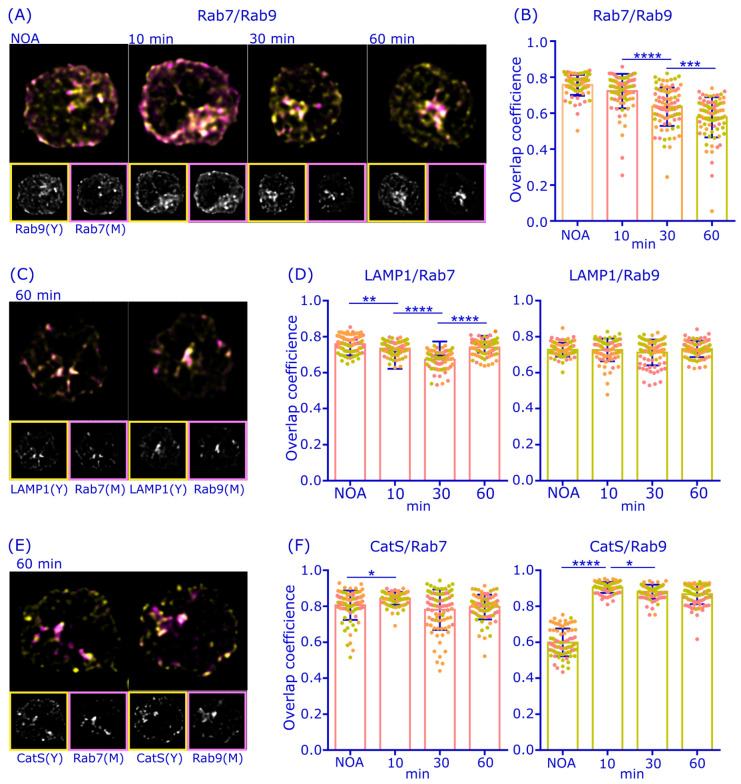
Rab7 and Rab9 show partially distinct behaviors in response to the antigen activation in B cells. A20D1.3 cells were either activated or not with 10 µg/mL α-IgM-647 (not shown) for 10, 30, or 60 min, before being fixed and processed for immunofluorescence analysis. The samples were imaged with 3D SDCM and deconvoluted with Huygens. (**A**) The samples were stained for anti-Rab7 (magenta) and anti-Rab9 (yellow) antibodies. (**B**) Overlap coefficient analyzed from samples in (**A**). (**C**) The samples were stained with either anti-Rab7 (magenta) or anti-Rab9 (magenta) and anti-LAMP1 (yellow) antibodies. The time point of 60 min is shown as an example. (**D**) Overlap coefficient analyzed from samples in (**C**). (**E**) The samples were stained with either anti-Rab7 (magenta) or anti-Rab9 (magenta) and anti-CatS (yellow) antibodies. The time point of 60 min is shown as an example. (**F**) Overlap coefficient analyzed from the samples in (**E**). In all panels (**B**,**D**,**F**), the data were analyzed from 20–32 cells from three individual experiments. Mean +/− SD is shown, dots represent cells (color coded for experiments). * *p* < 0.05, ** *p* < 0.01, *** *p* < 0.001, **** *p* < 0.0001 (one-way Anova). Image dimensions are 12 × 12 µm, planar images.

Next, we analyzed the colocalization of endogenous Rab7 and Rab9 in either activated or non-activated A20D1.3 B cells. Compared to primary B cells, these cells are slightly bigger and feature larger endosomes, which allows the usage of the spinning disc confocal microscope (SDCM) to acquire multiple images for quantitative analysis. Cells were allowed to internalize and process antigen for 10, 30, or 60 min. After activation, the samples were prepared for immunofluorescence analysis and imaged with the SDCM. As expected from the literature [32,33], and in agreement with our live imaging data, we detected the high colocalization of Rab7 and Rab9. The colocalization was strongest in the perinuclear area where Rab7 also strongly gathered over time whereas Rab9, additionally, showed a persistent peripheral localization (Figure 2A,B). The overlap coefficient of Rab7 and Rab9 was the highest in the non-activated cells and in the early activation time point (10 min) with the overlap of ≈75%. However, in agreement with the visibly distinct localization pattern, we detected a significant decrease in colocalization at later time points, largely due to the marked peripheral pool of small Rab9^+^ vesicles, indicating at least partially divergent roles for these two proteins upon antigen processing.

The antigen-containing vesicles in B cells typically colocalize with the late endosomal/lysosomal marker LAMP1, partially already at early stages of activation but especially in later time points [24]. We asked whether differential association with LAMP1 could explain the partial divergence between Rab7 and Rab9 and analyzed their colocalization with LAMP1 in different the time points of antigen activation. We saw a strong colocalization of LAMP1 with both Rab7 and Rab9 in non-activated cells as well as in different time points of activation. Rab9 showed highly stable colocalization through the different time points of activation, while Rab7-LAMP1 colocalization was more dynamic; however, it remained at high levels (Figure 2C,D).

Next, we compared the potential antigen processing capacity of the Rab7- or Rab9-enriched structures by examining colocalization with CathepsinS (CatS), the essential enzyme in preparing of the MHCII complexes for antigen peptide loading [1,10,11,12]. Interestingly, we found, that while Rab7-CatS colocalization stayed relatively constant and high in all time points (overlap coefficient ≈ 0.8), Rab9 showed clearly lower levels of colocalization in resting cells that then rapidly increased upon activation (overlap coefficient from 0.6 to 0.9) (Figure 2E,F). This suggests that Rab7 shows stronger residency in the MIIC type of vesicles that mature into antigen processing compartments. Together, these data indicate that, while both Rab proteins typically decorate the LAMP1^+^ late endosomal/lysosomal vesicles, these vesicle pools are also at least partially distinct. This differential behavior is notable in the rapid recruitment of Rab9 to the CatS^+^ vesicles upon cell activation and, on the other hand, in the dispersity of Rab9 compared to the Rab7 pool at the later stages of cell activation. Our data are also consistent with the literature that reports that although both Rab7 and Rab9 are involved with endosome maturation into late endosomes or lysosomes, they also play specific roles in the endosomal pathways [16,33,34,35,36].

### 3.2. Rab7 May Coordinate Perinuclear Antigen Gathering

**Figure 3:**
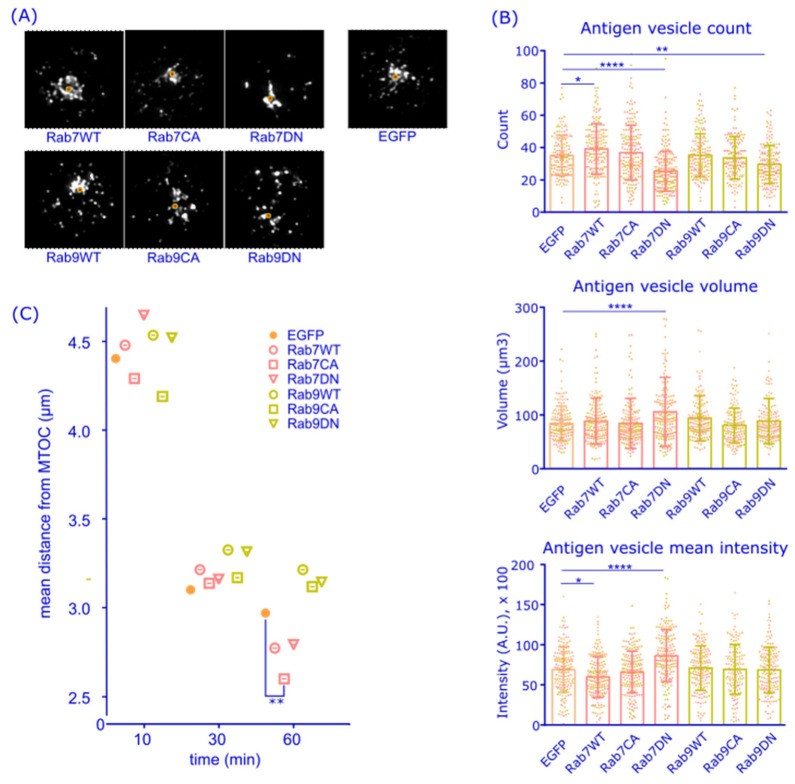
Antigen vesicle analysis in cells expressing GFP-Rab7 and -Rab9 constructs. (**A**) A20D1.3 cells transiently expressing the different EGFP-Rab7/Rab9 constructs were activated with α-IgM-555 (grayscale) for 60 min, fixed with PFA, and immunostained with anti-GFP (not shown) and anti-PCM-647 for MTOC (red circle). Cells were imaged with 3D SDCM and deconvoluted with Huygens. Representative cells expressing different Rab7/9 constructs are selected and antigen distribution is shown. The images are Z-stacks and the size is 12 × 12 µm. (**B**,**C**) Analysis of the GFP expressing cells in (**A**). (**B**) Antigen vesicle count, mean vesicle volume, and mean vesicle intensity per cell. Mean +/− SD is shown, dots represent cells (color coded for experiments). (**C**) Mean distance of the largest 10% of the antigen vesicles from the MTOC per cell is plotted. Antigen distribution in GFP+ cells was analyzed with a MATLAB script [24] from >20 cells from four individual experiments. One-way Anova, compared to EGFP. * *p* < 0.05, ** *p* < 0.01, **** *p* < 0.0001.

Next, we questioned whether Rab7 and Rab9 activity is required for antigen trafficking to the perinuclear MIIC. We utilized commonly used constitutively active (CA) and dominant negative (DN) point mutations of Rab-family GTPases that are expected to lock the proteins in GTP- or GDP-bound states, respectively [37,38,39]. We transiently expressed different EGFP-tagged versions of Rab7 or Rab9, or EGFP only, in A20D1.3 B cells. The cells were activated with fluorescent α-IgM for 60 min and subjected to immunofluorescence analysis including staining for the microtubule organization center (MTOC) (Figure 3A). We run a detailed analysis of the antigen vesicle properties and intracellular distribution (Supplementary Figure S2A) in all the over-expression conditions. Particle analysis showed that particularly cells expressing Rab7DN, but also those expressing Rab9DN, featured reduced numbers of antigen vesicles than control cells (Figure 3B). The expression of Rab7DN also led to enlarged antigen vesicle volumes and increased vesicle brightness. The overexpression of Rab7WT led to the opposite features, namely increased numbers of antigen vesicles with reduced brightness. On the other hand, none of the constructs induced detectable changes in the total antigen fluorescence intensities, as measured by microscopy (Supplementary Figure S2B) or in the overall capability to internalize antigen, as studied by flow cytometer (Supplementary Figure S3A). We then decided to concentrate on the migration of the largest 10% of the antigen vesicles, as the smaller vesicles are often retained in the cell periphery while the larger ones are found close to the MTOC [24]. Thus, we examined the distance of the largest 10% of antigen vesicles from the MTOC over time. We noticed that, 60 min after the activation, Rab7CA-expressing cells showed a significantly tighter accumulation of the antigen vesicles towards the MTOC as compared to control cells (2.6 µm vs. 3.0 µm, respectively) (Figure 3C). To illustrate the vesicle distribution better, we plotted the distance distributions of the largest 10% of the antigen vesicles 60 min after activation (Supplementary Figure S2C). Rab7CA-expressing cells featured 48% of the large antigen vesicles within a distance of 2 µm from the MTOC, whereas in control cells, the corresponding portion was 40%. Together, these findings promote the role of Rab7 over Rab9 in antigen trafficking to the perinuclear region.

### 3.3. Antigen Processing Activity Prefers Rab7^+^ over Rab9^+^ Vesicles

**Figure 4:**
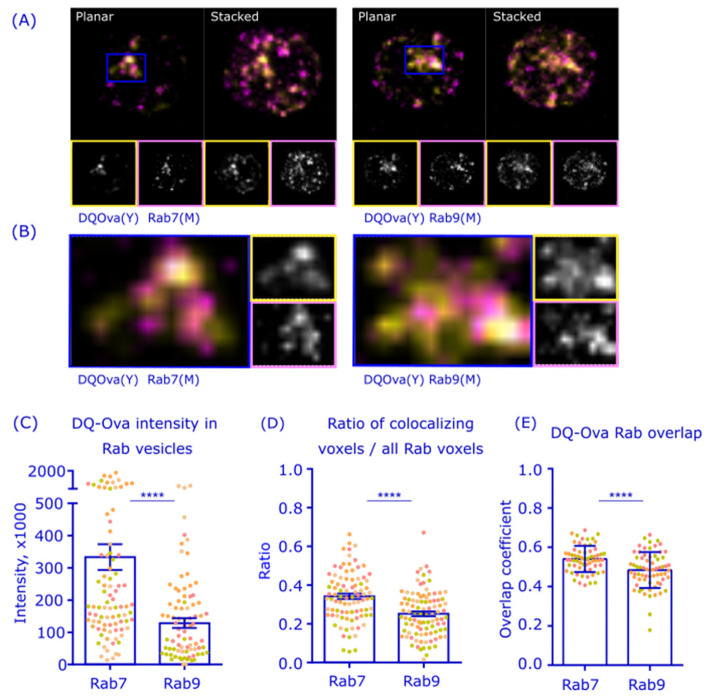
Antigen degradation, measured by DQ-Ova signal, prefers Rab7^+^ vesicles. (**A**) A20D1.3 cells were activated with DQ-Ova-HEL (yellow) for 60 min, PFA fixed and immunostained with anti-Rab7 (magenta) or anti-Rab9 (magenta) antibodies. Cells were imaged with 3D SDCM and deconvoluted with Huygens. Planar and stacked images are shown with image sizes of 12 × 12 µm. (**B**) Zoomed-in images from the blue boxes in (**A**). (**C**–**E**) Analysis from the data in (**A**): particle analysis to determine the total DQOva intensity in Rab7^+^ and Rab9^+^ positive vesicles (**C**), analysis of the ratio of Rab-colocalizing DQ-Ova voxels to all Rab voxels (**D**), and colocalization of DQ-Ova and Rab analyzed by Overlap coefficient (**E**). Data was analyzed from >20 cells from four individual experiments. Mean +/− SEM is shown, dots represent cells (color coded for experiments). **** *p* < 0.0001 (unpaired *t*-test).

In addition to the influence of Rab7 constructs in antigen vesicle positioning, we saw a significant increase in antigen intensity in cells expressing Rab7DN (Figure 3B). This provoked us to further study antigen processing or degradation capacity in vesicles positive for Rab7 or Rab9 in non-transfected cells. To this end, we utilized a degradation-sensitive probe DQ-Ovalbumin (DQ-Ova). DQ-Ova becomes fluorescent only when its folded three-dimensional structure, that keeps the BODIPY fluorophores quenched, is lost [40]. In this assay, we activated the B cells with biotin-linked antigen hen egg lysozyme (HEL), that is specific for the D1.3 IgM receptor, sandwiched to the biotinylated DQ-Ova via streptavidin. After 60 min of activation, cells were fixed, immunostained for endogenous Rab7 or Rab9, and imaged in 3D with SDCM (Figure 4A). We measured the intensity of the DQ-Ova signal in Rab7^+^ and Rab9^+^ vesicles by performing particle analysis on the images. The data revealed almost three times higher levels of the DQ-Ova signal in Rab7^+^ vesicles as compared to Rab9^+^ vesicles (Figure 4C). In line with this, the analysis of the ratio of the Rab-colocalizing DQ-Ova voxels to all Rab voxels reported a higher proportion Rab7 than with Rab9 in compartments of Ag degradation (0.34 vs. 0.25; Figure 4D) and the overlap coefficients of the DQ-Ova signal were also significantly higher with Rab7 than with Rab9 (0.54 vs. 0.48; Figure 4E).

Notably, when inspecting the images, it was seen that some of the DQ-Ova signal also appeared outside of the Rab7/9^+^ vesicles. This was most visible in the central cluster, where the highest DQ-Ova signal showed only partial correlation with either of the Rab proteins (Figure 4B). Interestingly, these data suggested that, although antigen degradation preferred Rab7^+^ over Rab9^+^ vesicles, there could be yet another diversion of degradative vesicles, from the Rab7^+^ to the adjacent compartments, when the antigen is gathered close to the MTOC.

### 3.4. Antigen Is Gathered in LC3^+^ and H2-M^+^ Perinuclear Compartments

**Figure 5:**
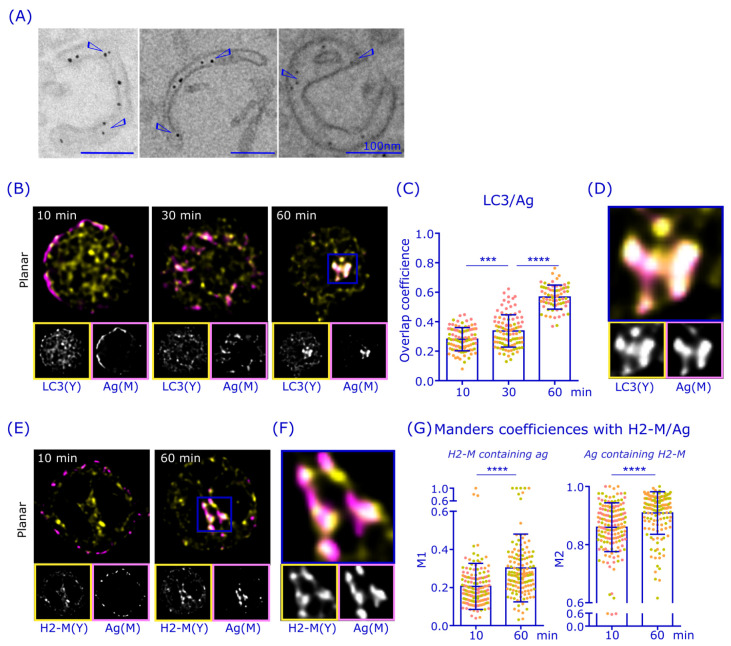
Antigen colocalizes with LC3 and H2-M in the perinuclear region. (**A**) A20D1.3 B cells were activated with 6 nm gold-conjugated α-IgM for 75 min, after which they were processed for TEM. Examples of antigen-gold in phagophore-like structures. Scale bar 100 nm. (**B**) A20D1.3 cells were activated with 10 µg/mL α-IgM-647 (magenta) for 10 min, 30 min, and 60 min, PFA fixed and immunostained with anti-LC3 antibodies (yellow). Cells were imaged with 3D SDCM and deconvoluted with Huygens. Image size 12 × 12 µm, planar images. (**C**) Overlap coefficient of the data in (**B**). Analyzed from >20 cells from three individual experiments. Mean +/− SD is shown and dots represent cells (color coded for experiments). *** *p* < 0.001, **** *p* < 0.0001 (one-way Anova) (**D**) Zoomed image from the blue box in (**B**). (**E**) A20D1.3 cells were activated with α-IgM-647 (magenta) for 10 min and 60 min, fixed and permeabilized with methanol-acetone and immunostained with anti-H2-M (yellow). Cells were imaged with 3D SDCM and deconvoluted with Huygens. Planar images are shown, with image size of 12 × 12 µm. (**F**) Zoomed image from the blue box in (**E**). (**G**) Manders colocalization coefficients M1 and M2 of the data in (**E**). LC3 was analyzed from 19–32 from three individual experiments and H2-M from 44–58 cells from three individual experiments. Mean +/− SD is shown, dots represent cells, color coded for experiments. **** *p* < 0.0001 (unpaired *t*-test).

The finding above, namely that the highest DQ-Ova activity in the perinuclear area seemed to reside in membrane domains adjacent to Rab7 (Figure 4B), prompted us to utilize an electron microscope (EM) to visualize the structures of antigen-containing endosomal compartments. We activated cells with nanogold-conjugated α-IgM for 75 min and processed the samples for transmission-EM (TEM) imaging [24]. While antigen was found in endosomes showing high structural heterogeneity (Supplementary Figure S4A), interestingly, in several examples, we also found antigen in phagophore-like compartments (Figure 5A, Supplementary Figure S4B). Macro-autophagosomes (hereafter autophagosomes) have different shapes depending on their maturation state. While phagophores have their renown structure of a double-membraned cup, autophagosomes are double-membraned rings [41,42]. Furthermore, while Rab7 is best known for its role in vesicle transport and endosomal maturation, it regulates multiple aspects of cellular vesicles, including autophagosome maturation and fusion with lysosomes [43,44,45,46,47]. These unexpected findings led us to speculate that some of the antigen could be processed in autophagosome-related structures.

We proceeded to investigate the potential role of autophagy in antigen processing by analyzing the colocalization of the autophagosome-linked protein LC3 with antigen by immunofluorescence (Figure 5B). A20D1.3 cells showed a strong signal for LC3, which exhibited a partial colocalization with antigen already 10 min after the activation, although a large fraction of LC3 was still scattered in the cytosol. Importantly, the antigen-LC3 colocalization markedly increased over time, reaching an overlap coefficient of 0.6 at 60 min after activation despite the clear remaining population of LC3 that did not become associated with antigen (Figure 5B,C). Interestingly, when we inspected the perinuclear antigen cluster in more detail, we saw a striking colocalization of LC3 and antigen (Figure 5D). These data suggest that autophagosome-linked protein machinery could play an interesting role in antigen processing, particularly in the long-lasting antigen processing compartment in the perinuclear region.

To investigate whether the perinuclear antigen-LC3^+^ compartments could be functional in antigen processing and pAg loading, we next examined the localization of H2-M, the chaperone molecule essential for peptide antigen loading onto MHCII. As expected, H2-M showed very strong visual colocalization with the antigen, especially in the perinuclear region (Figure 5E,F), to very similar degree to that of LC3. Due to the relatively broad localization of H2-M throughout the cells, we utilized two-way Manders colocalization coefficient analysis to analyze the colocalization of antigen and H2-M. This analysis revealed a very high localization of H2-M in the antigen vesicles (M2) already at 10 min after activation and even stronger colocalization at 60 min after activation, when nearly all the antigen vesicles (≈91%) contained H2-M (Figure 5G), resembling the behavior of LC3. Together these findings indicate that the perinuclear antigen cluster is likely to contain both LC3 and the pAg loading machinery H2-M.

### 3.5. Disturbing Rab7 and Autophagosome Functions Inhibit Peptide Antigen Presentation

**Figure 6:**
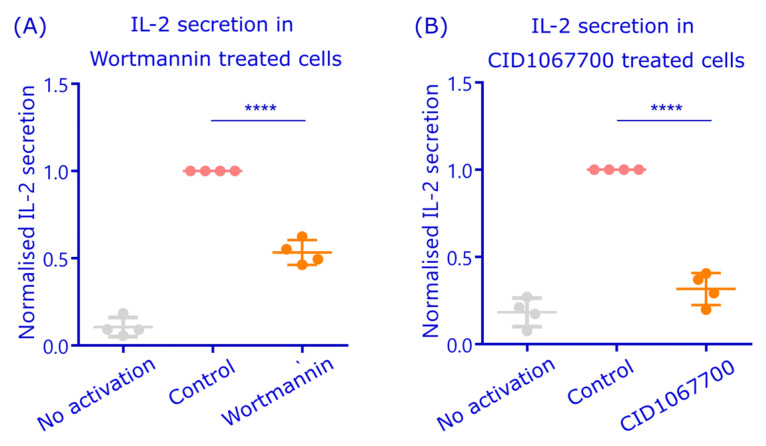
Inhibitors against Rab7 and autophagosome maturation inhibit peptide antigen presentation. (**A**) A20D1.3 cells were treated with 1 µM Wortmannin at the same time as they were activated or not with 5 µg/mL of HEL for 1 h, washed, and then mixed with 1E5 T cells for 17 h. The IL-2 secretion was measured by ELISA and normalized to the control cells. (**B**) The antigen presentation assay was performed as in (**A**) but the samples were continuously treated with 60 µM CID1067700 to inhibit the Rab7 activity through the whole experiment due to the reversible nature of the drug. Analyses are from 4 individual experiments (dots), normalized to the control. **** *p* < 0.0001 (unpaired *t*-test).

As pAg-MHCII presentation efficiency can be determined by B cell capability to activate cognate T cells, we sought for an experimental setup to test the function of autophagy machinery and Rab7 in antigen processing and pAg presentation in B cells. The 1E5 hybridoma T cells, which are specific for a HEL-derived peptide, can be activated with A20D1.3 cells stimulated with HEL. T cells then secrete IL-2, the level of which can be determined by ELISA assay, for example. To inhibit autophagy, we utilized the pharmacological inhibitor Wortmannin. Wortmannin has been widely used as an inhibitor for autophagy as it inhibits phosphatidylinositol-3-phosphate (PI_3_P) formation through PI3K [48]. PI3P, on the other hand, is a specific lipid molecule for autophagy membranes [49] and it is essential for the formation of the autophagosome precursor formation [50]. Indeed, the IL-2 secretion by T cells in the pAg presentation assay was dropped approximately to half the normal level upon Wortmannin treatment (Figure 6A). The antigen internalization was found to be normal in Wortmannin-treated cells (Supplementary Figure S3B), indicating that the phenotype would not be derived from a general diminution of BCR signaling, that also triggers the PI_3_K pathway.

Finally, we used a pharmacological inhibitor CID1067700, a Ras-family GTPase-inhibitor, that binds Rab7-GTP with very high affinity and prevents its switch into the active state [51]. Despite the ability to inhibit also several other GTPases in vitro, in B cells, CID1067700 has been reported to particularly function via affecting Rab7 [26,51]. When we inspected the CID1067700-treated cells, they were normally viable and internalized antigen (Supplementary Figure S3B). Yet, in the antigen presentation assay, CID1067700 drastically decreased the IL-2 secretion from the activated T cells to only 20% of the non-treated samples (Figure 6B).

Together, our data suggest that pAg presentation and T cell activation is at least partially regulated by Rab7. Moreover, the autophagosome-related structures could also play an important role in B cell antigen processing as the treatment with Wortmannin also led to decreased pAg presentation. Considering the strong perinuclear colocalization of LC3 and H2-M with antigen, and the adjacent localization of Rab7 as well as its role in perinuclear antigen trafficking, we propose a model where Rab7 orchestrates vesicular pathways for antigen processing, and these pathways could be at least partially connected to autophagy-related machineries to generate the final perinuclear MIIC.

## 4. Discussion

B cells are known to have a sophisticated and specialized vesicular network for antigen processing [24,52]. In this work, we aimed to increase our understanding of how antigen targets the dense perinuclear antigen compartment, which is likely the major MIIC that is essential for long-lasting pAg presentation. We found that, while both vesicle traffic regulators, Rab7 and Rab9, showed high colocalization with each other as well as with internalized antigen throughout its migration towards the perinuclear region, the correlation of signals in the perinuclear MIIC area was not particularly high. Instead, we found that antigen, when clustered in the perinuclear region, showed very strong colocalization with LC3, an autophagosomal protein, as well as with H2-M, an essential factor for MHCII pAg loading. Taken together, our various microscopic approaches, pharmacological inhibitors, and pAg presentation assays propose a role for Rab7 in B cell antigen processing. We postulate that autophagy-related membrane compartments could also be involved in antigen processing and the possible interplay between Rab7 and these compartments is an interesting topic for future studies.

By visualizing endogenous Rab7 and Rab9 with 3D SDCM, accompanied by the deconvolution of the images to improve the resolution, we detected high levels of colocalization between Rab7 and Rab9; however, with a decrease over time after BCR activation (Figure 2A,B). In consensus with the literature, Rab7 and Rab9 were both found in late endosomes marked with LAMP1, and the levels of colocalization remained high during the 60 min activation time (Figure 2C,D). Thus, it appears that, during B cell activation, Rab7 and Rab9 partially divert into distinct endolysosomal pools as described, as also, for example, shown by Barbero et al. for epithelial cells [36,53]. We also found CatS, a critical enzyme required for trimming the MHCII invariant chain enabling pAg loading, to colocalize strongly with both Rab7 and Rab9 in activated B cells (Figure 2E,F). Interestingly, however, the colocalization of Rab9 and CatS was at lower levels in resting cells and clearly triggered by antigen activation, further suggesting the overlapping yet separate functions of Rab7 and Rab9 in relation to antigen processing. However, due to the small cytoplasmic space and vesicle size in B cells, the resolution of light microscopy often becomes limiting and colocalization analysis should also be interpreted with caution [54]. Testing the idea of whether vesicular compartments, or vesicle domains, with differential Rab7 or Rab9 could also behave differently in antigen degradation, we labeled antigen with DQ-Ova, which generates the fluorescent signal only upon degradation, and indeed found a significantly brighter fluorescence in Rab7^+^ endosomes compared to Rab9^+^ counterparts (Figure 4). This was also in line with the observation that the expression of the non-functional Rab7DN mutant led to the Ag-vesicles of increased size and fluorescence, which was not seen with the comparable Rab9 DN mutant (Figure 3). These findings supported the view that the antigen associated with Rab7^+^ vesicles would have a higher degradation capacity. This also fits well with the literature describing Rab7 as a key player in cargo degradation and coordinating degradation steps in various cellular systems [37,47].

The critical outcome of B cell antigen processing is pAg presentation on MHCII to the cognate T cells. With a system of HEL-specific A20D1.3 B cells and 1E5 T cell hybridomas recognizing HEL-derived pAg, we were able to measure antigen presentation in coculture settings. Importantly, we showed that T cell activation was blocked when the cells were treated with a pharmacological inhibitor of Rab7 CID1067700 (Figure 6). Although CID1067700 has nanomolar Ki values and reportedly possesses the highest inhibitor potency for Rab7, it can affect other GTPases too [51]. However, the CID1067700-induced reduction in B cell class switch recombination was shown to specifically originate from the effect on Rab7 [26], indicating that, in B cells, Rab7 would present the main target for this drug. Notably, the CID1067700-treated cells were taking up antigen in a normal manner, indicating that, in the conditions used, the inhibitor does not affect BCR signaling or the general fitness of the cells. Unfortunately, the generation of stable cell lines expressing different active or inactive mutants of Rab7 and Rab9, to functionally test their role in antigen presentation, was not successful, which was most likely due to the harmful effects of Rab overexpression on cell homeostasis. As the antigen presentation assay measures the end result of antigen processing, T cell activation, the details of the particular steps affected cannot be deduced from these data. As Rab7 has several important roles in endosomal trafficking and processing [47], it is plausible that Rab7 inhibition functions at many levels. Based on our data on Rab7 localization (Figure 2), its functional role in antigen vesicle trafficking (Figure 3 and Supplementary Figure S2), the correlation with antigen degradation (Figure 4), as well as our previous studies showing Rab7 already in the eMIICs [24], we postulate that Rab7 functions at various levels, taking part in the maintenance of the vesicles’ degradative capacity as well as in the vesicle traffic towards perinuclear MIIC.

The partial localization of antigen in autophagosome-like vesicular structures, as suggested by TEM (Figure 5; Supplementary Figure S4), prompted us to also study the well-known autophagy marker LC3. Interestingly, we detected a strong colocalization of antigen with the LC3 and the MHCII peptide loading chaperone H2-M in the perinuclear region (Figure 5). To functionally test whether autophagosomal machinery could be involved in pAg presentation, we used Wortmannin, an PI3K inhibitor that also strongly inhibits autophagy and found a significantly decreased pAg presentation in the cognate B-T cell co-culture assay (Figure 6). Notably, the autophagosome maturation can be guided by Rab7, as, for instance, the Rab7DN expression was reported to fully block autophagosomal degradation [55,56,57], also providing a plausible connection between Rab7 and autophagosomal pathways in antigen processing.

Autophagy is classically described as a conserved degradative pathway that cleans the cytoplasm from damaged or superfluous organelles and protein aggregates [58] and, thus, overlapping with the external antigen processing pathway seems rather surprising. Nevertheless, internalized antigen has also been found to be present in atypical, ring-shape autophagic structures in B cells in a recent study, where the non-canonical autophagy was linked to mitophagy and metabolic changes that were critical for B cell differentiation [59]. Various B cell functions have been shown to depend on autophagy as, for instance, antibody producing plasma cells and long lived memory B cells possess very high autophagic activity, and autophagy is reported to control the number and immunoglobulin secretion of memory B cells [58,59,60]. Also, a role for autophagy in antigen presentation has been demonstrated in other APCs, where there is continuous material transfer from autophagosomes to the MIIC by the fusion of these compartments [61,62,63]. It has also been shown that BCR activation induces autophagy in B cells and the role of autophagosomes in the presentation of non-BCR specific antigens, namely cytosolic or viral antigens, has been demonstrated [64]. Notably, autophagy-linked protein machineries are also found to have various so-called non-canonical functions, highlighting that not every vesicle with an autophagy-linked protein is a classical autophagosome [65]. Importantly, the non-canonical function of an autophagy-linked protein in B cell activation is ATG5. which was recently shown to play a role in the vesicular targeting of BCR-antigen complexes to MIIC and the presentation of antigens encountered in particulate form [66]. As classical autophagosomes are dedicated to clear and destroy unnecessary cytosolic content [67], it would appear likely that the possible role of autophagy in antigen processing would be non-canonical.

## 5. Conclusions

In this work, we studied the regulation of Ag trafficking and processing by Rab 7 and Rab9 small GTPases in B cells. Our results show Rab7 to exhibit higher overlap with antigen, MIIC components, and antigen degradation than Rab9. A functional role of Rab7 in Ag processing is promoted by the increased Ag vesicle size and intensity upon the expression of Rab7DN and the decreased pAg presentation upon the pharmacological inhibition of Rab7. We also detected the strong colocalization of perinuclear antigen clusters with autophagy protein LC3 and found that the pharmacological inhibition of autophagy also inhibited pAg presentation. Together, our study proposed that both Rab7^+^ and autophagy-linked machinery are involved in B cell antigen processing and presentation. Dissecting the complex vesicular network of antigen processing requires more studies with an appreciation of the potential cross-talk between endosomal and autophagy compartments.

## Data Availability

The original contributions presented in the study are included in the article and Supplementary Material; further inquiries can be directed to the corresponding author.

## Supplementary Materials

The following supporting information can be downloaded at: https://www.mdpi.com/article/10.3390/cells12212566/s1, Figure S1: Supporting information for Figure 1, and live imaging of Rab7 and Rab9; Figure S2: The antigen vesicle distances from the MTOC; Figure S3: Antigen internalization; Figure S4: Antigen localization in phagophore-like compartments; Video S1: A20D1.3 cells expressing EGFP-Rab7 and mCherry-Rab9; Video S2: A20D1.3 cells expressing EGFP-Rab7 and mCherry-Rab9 activated with antigen.
