# Supplementary material for: The Small GTPase Rab7 Regulates Antigen Processing in B Cells in a Possible Interplay with Autophagy Machinery
Source: Cells. 2023 Nov 2;12(21):2566. doi: 10.3390/cells12212566 (PMC10649364; doi:10.3390/cells12212566)
Supplement: Supplementary file 1 [file cells-12-02566-s001.zip › Supplementary material Runsala_SUBMIT.pdf]

The small GTPase Rab7 regulates antigen processing in B cells in a possible interplay with autophagy machinery

Marika Runsala[1,2,3], Elina Kuokkanen[1], Eveliina Uski[1], Vid Sustar[1], M. Özge Balci[1,2,3], Johanna Rajala[1], Vilma Paavola[1] and Pieta K. Mattila[1,2,3]*

SUPPLEMENTARY MATERIALS

Supplementary Video S1.
A20D1.3 cells transiently expressing both EGFP-Rab7 (yellow) and mCherry-Rab9 (magenta) were imaged live with SDCM. The cells were imaged for 5 minutes with 806 ms frame capturing speed.

Supplementary Video S2.
A20D1.3 cells transiently expressing both EGFP-Rab7 (yellow) and mCherry-Rab9 (magenta) were activated with AlexaFluor647-labelled αIgM F(ab')$_2$ fragments (cyan). The cells were imaged live with SDCM for 5 minutes starting at 25 minutes after the activation, with 980 ms frame capturing speed.

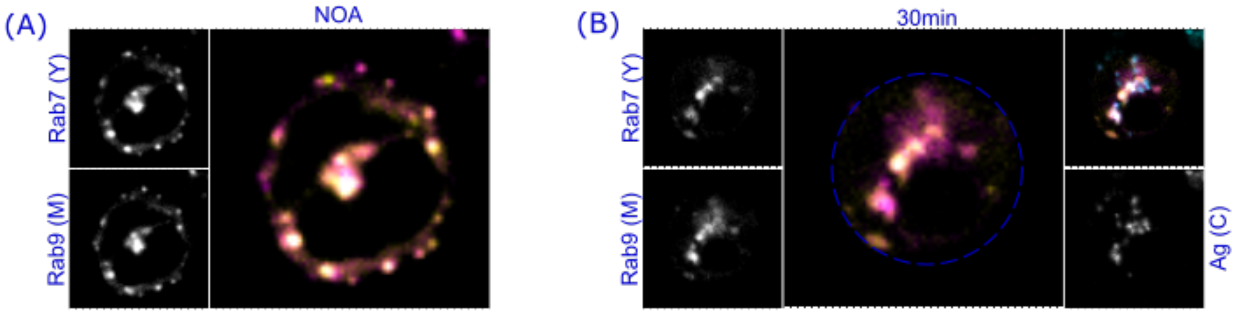

Supplementary Figure S1. The localization of Rab7 and Rab9.

A20D1.3 cells transiently expressing both EGFP-Rab7 (yellow) and mCherry-Rab9 (magenta) were imaged live with SDCM. **(A)** The cells were either left non-activated or **(B)** activated for 30 minutes with AlexaFluor647-labelled αIgM F(ab')$_2$ fragments (cyan). Imaging was carried out with 3D SDCM and representative planar images are shown.

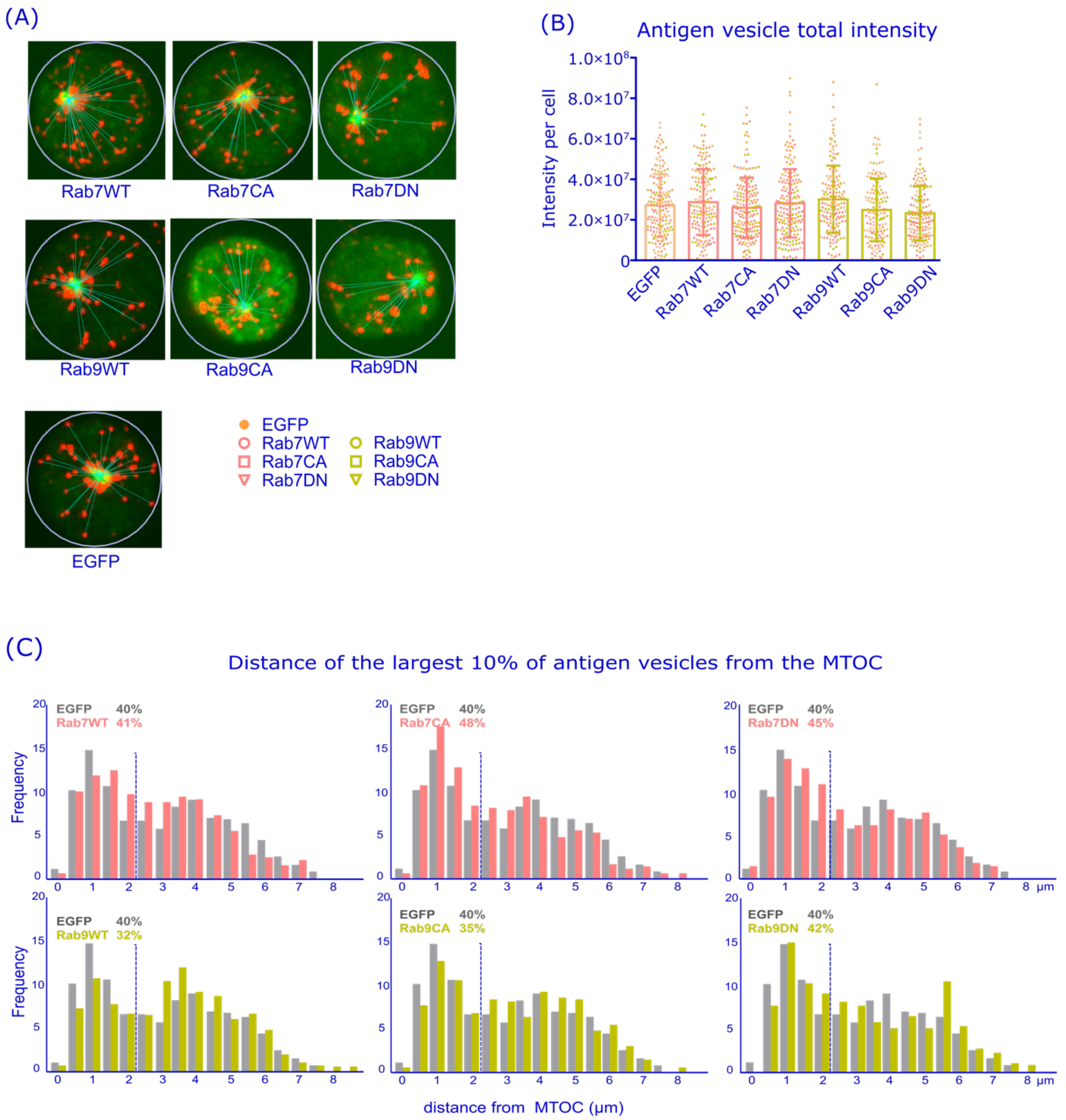

Supplementary Figure S2. The antigen vesicle distance from the MTOC.

(A) A20D1.3 cells transiently expressing the different EGFP-Rab7/Rab9 constructs were activated with αIgM-555 (red) for 60 min, PFA fixed and stained with anti-GFP (not shown) and anti-PCM-647 for MTOC (green). Cells were imaged with 3D SDCM and deconvoluted with Huygens. Green lines represent distance measurements in the following particle analyses (B,C; and Figure 3B,C). Square 10x10 µm, stacked Z-projections. **(B)** Total antigen intensity in the cells expressing different constructs. Data analyzed from >20 cells from four individual experiments. Mean +/- SEM shown, dots represent cells (color coded for experiments). **(C)** >2500 vesicles were pooled from the cells in (B). From the pooled vesicles, largest 10% (>250 vesicles) were taken to the analysis and their distance from the MTOC (green lines in (A)) was determined with a MATLAB script (Hernández-Pérez et al. 2020). The data was binned on histograms with 0.5 µm bin width (C).

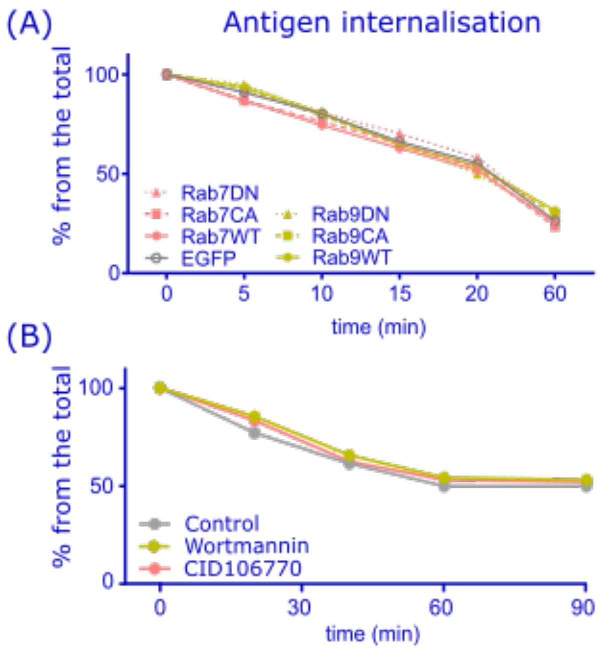

Supplementary Figure S3. Antigen internalization.
(A) A20D1.3 cells transiently expressing the different EGFP-Rab7/Rab9 constructs were labelled with biotinylated αIgM and incubated at 37 ºC for different time points, after which they were stained on ice with AlexaFluor488-streptavidin to detect surface-resident αIgM. The intensity was normalised to time point 0. The data is a mean from three independent experiments. **(B)** A20D1.3 cells were treated with 1 µM Wortmannin or 60 µM CID106770, or DMSO control were treated and analysed as in (A). The data is from one experiment.

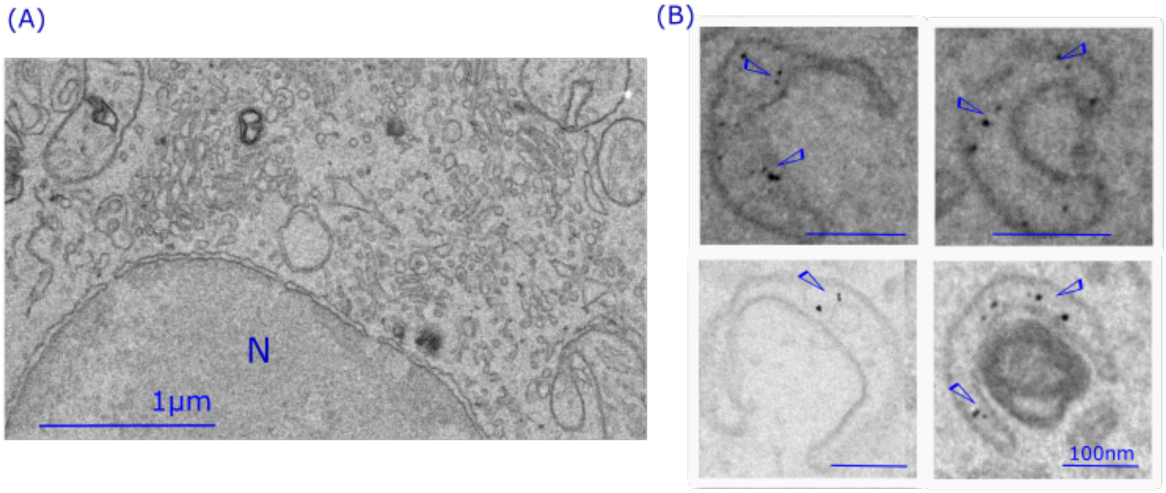

Supplementary Figure S4. Antigen localization in phagophore –like compartments
A20D1.3 B cells were activated with αIgM conjugated with 6 nm gold particles for 75 minutes after which they were processed for TEM. **(A)** Divergent endosomes in the perinuclear area (N = nucleus). Scale bar 1 µm. **(B)** Gold-conjugated antigen (arrows) in phagophore-like structures. Scale bar 100 nm.
